# P35 Lupus masquerading as Cholangiocarcinoma: One of the many faces of multifaceted lupus

**DOI:** 10.1093/rap/rkac067.035

**Published:** 2022-09-28

**Authors:** Bilal Tarar, Subhra Raghuvanshi

**Affiliations:** Haywood hospital, Stoke on trent, United Kingdom; Wrexham Maelor hospital, Wrexham, United Kingdom

## Abstract

**Introduction/Background:**

Systemic lupus erythematosus (SLE) is a complex autoimmune condition which can present with a wide variety of clinical features. This variation can lead to delayed diagnosis or misdiagnosis. Gastroenetrological pathology in SLE is rare but primary autoimmune hepatitis, pancreatitis, lupus enteritis, cholestatic biliary disease and protein losing enteropathy have all been reported. We would like to share the first known presentation of SLE as cholangiocarcinoma.

**Description/Method:**

A 62-year-old, previously fit and well, gentleman presented with a 1 month history of painless jaundice, a palpable gallbladder (Courvoisier syndrome), dark urine, pale stool, generalised rash, pruritus, weight loss and anorexia. The presence of these red flag features raised concerns. Initial investigation with Endoscopic retrograde cholangiopancreatography (ERCP) revealed distal common bile duct (CBD) stricture which was further explored with computerised tomography of thorax, abdomen, pelvis and magnetic retrograde cholangiopancreatogram (MRCP) both raising the suspicion of a cholangiocarcinoma in the presence of constitutional symptoms. Several biopsies and brushings taken during multiple ERCP’s failed to identify clear mitotic cells, IgG 4 staining remained negative. Further investigation with Positron emission tomography – computerised tomography (PET-CT) showed significant FDG uptake around the upper part of the stent with final working diagnosis of Cholangiocarcinoma and a Whipple’s procedure was planned.

In the interim, the patient developed swollen painful hands, scleritis and a yellow blistering rash. He was referred urgently onto the Rheumatology team after receiving the positive immunology results: ANA (1:1280), Anti double stranded DNA and Rheumatoid Factor (RF) along with hypocomplementemia (C3&C4).

**Discussion/Results:**

Subsequent work up under rheumatology was done considering the possibilities of diagnosis of Systemic lupus erythematosus (SLE), Lupus related autoimmune hepatitis and reactive arthritis as a result of underlying malignancy. Patient’s immunology and other clinical features such as skin rash were strongly supportive of SLE as a primary diagnosis. He was initially treated with low dose prednisolone with dramatic improvement in arthritis and gradual improvement in liver function test. Following MDT discussions with the hepato-biliary team it was felt that lupus was the cause of the biliary stricture which settled well with steroid treatment. A final ERCP was arranged to remove the stent and take final brushings. Methotrexate was started at this point. His SLE remains well controlled on methotrexate and his liver function tests, including bilirubin, remain within the normal range. Patient has now been completely asymptomatic and is discharged from the care of hepatobiliary team with long term follow up for his SLE with rheumatology.

**Key learning points/Conclusion:**

This is extremely rare first presentation of SLE presenting with painless jaundice and biliary stricture which improved both biochemically and clinically with treatment of SLE. The key learning points included:

Urgent review by rheumatology can facilitate timely diagnosis and preventable admissions/interventions such as major intraabdominal procedure in the form of whipple’s disease planned in our case.

Cancer remains a major cause of mortality and morbidity in rheumatological conditions like SLE. Hence initial diagnosis can become extremely difficult if an overlap between these two happens.

Treatment options can be limited initially and steroids remain the main stay of treatment till mitotic lesions have been ruled out and disease modifying agents can be used without any contraindications. But disease control for our conditions remains challenging in presence of cancers.

